# Workshops as a qualitative research method in health research

**DOI:** 10.1136/bmjopen-2025-106459

**Published:** 2025-09-26

**Authors:** Alicia O’Cathain

**Affiliations:** 1 Sheffield Centre of Health and Related Research, University of Sheffield, Sheffield, UK

**Keywords:** STATISTICS & RESEARCH METHODS, QUALITATIVE RESEARCH, Health Services

## Abstract

**Abstract:**

**Aim:**

Researchers are increasingly using workshops within health research, particularly in the context of developing complex interventions. The status of workshops within health research is not clear. Are workshops a research method or a form of stakeholder involvement akin to patient and public involvement? Do they require ethics approval? How are data collected and analysed? How should the results be published – if at all?

**Methods:**

Reflection on the methodological literature.

**Reflections:**

Researchers can frame workshops as a qualitative research method if they aim to generate new knowledge that is useful to stakeholders external to their research project and therefore aim to publish the findings of the workshops. In that context, ethics approval is required, with written informed consent taken from participants. Data collection can occur using a range of approaches including post-it notes, handwritten notes or audio or video recordings of discussions. Data can be analysed using a range of approaches including thematic or content analysis. Like any qualitative research, results can be published in a research article. A list of issues to consider and report when undertaking workshops as a research method is offered, based on methodological literature from a range of research fields. Alternatively, researchers can frame workshops as ‘stakeholder involvement in research’ if they aim to identify knowledge for use within their research project only. The product of these workshops might be characterised as a set of actions for the research team to take. Formal analysis will not be necessary—merely identification of actions—and reporting within publications may be similar to the reporting of patient and public involvement activity with a research project. Researchers may face grey areas when deciding which route to adopt. Team reflection and documenting the justification for the decision made may help to formulate appropriate decisions.

## Introduction

 A workshop involves bringing a group of people together to discuss an issue. Workshops are increasingly being used in health research (health services research, health technology assessment, public health) particularly in the context of developing complex interventions or developing dissemination strategies for research projects. In these contexts, a range of stakeholders are brought together for one or multiple workshops to consider the evidence base for an intervention, identify the need for an intervention, describe the context in which an intervention might operate and propose the required components of an intervention that could address identified needs^1^ or consider the research project findings so the research team can maximise the impact of their research.

### Are workshops a research method?

The status of workshops within health research is not clear. Research methods are systematic approaches to collect and analyse data to identify new knowledge or better understanding of an issue. Workshops can fit this definition of a research method yet are not always listed as a research method in well-known research methods books.^2 3^ Being clear that workshops are a research method can give them credibility. This might help researchers to use workshops rather than other research methods which they might consider to be more publishable. For example, researchers might use consensus exercises such as Delphi exercises to identify the components or properties of an intervention when this might stymie the debate and disagreement that needs to be aired when developing interventions. Or researchers might use focus groups when these are not appropriate. Trettin *et al* articulate the differences between workshops and focus groups.^4^ They describe workshops as aiming to include heterogeneous participants, often involving large numbers of participants, allowing for the unpredictable and sometimes having multiple sequential workshops. In contrast, they describe focus groups as aiming to include homogeneous participants, involving small numbers of participants, being structured and predictable and being one-off events. It may also be the case that claiming workshops as a research method may place onus on researchers to formalise the conduct, analysis and reporting of them—and indeed publish the findings from them—when this may not be appropriate for the aim of those workshops.

The proposal set out here is that researchers can decide how to frame their workshops by considering the purpose of those workshops. Researchers can frame workshops as a qualitative research method if they aim to generate new knowledge that is useful to those external to their research project. They would aim to publish findings in detail to contribute to the evidence base. In this context, ethics approval is required, with written informed consent taken from participants. Data collection may occur using a variety of approaches including post-it notes, handwritten notes or audio or video recordings of discussions. Data may be analysed using a variety of approaches including thematic or content analysis. A qualitative research article may be published to report findings. Alternatively, researchers can frame workshops as ‘stakeholder involvement in research’ to shape their research project. Workshop participants are akin to patient and public involvement members where they shape an ongoing or future research project or an intervention in its early stages of development.^5^ The product of these workshops might be a study design for a future research project, an overview of intended components of an intervention or a dissemination and impact strategy at the end of a research project. The product might be characterised as a set of actions for the research team to take. Formal analysis will not be necessary—merely identification of actions. This activity may be described briefly within wider reports and publications from the research project—akin to using the Guidance for Reporting Involvement of Patients and the Public, version 2 (GRIPP2) when describing patient and public involvement in a research project^6^—but would not be the basis for a standalone publication. Drawing a distinction between these two framings may not be easy in some circumstances. Team reflection and documenting the justification for the decision made may help to formulate appropriate decisions.

The aim of this methodological reflection is to help to improve the credibility of workshops as a research method within the health research community so that researchers feel more comfortable including them in grant applications and peer-reviewed journal articles. The remaining part of this paper describes key issues for researchers to consider when planning and reporting workshops as a research method.

## Methods

Researchers in disciplines outside health research have described workshops as a research method and identified the key issues that would need to be considered or reported when undertaking workshops as a research method, including: business research,^7^ educational research,^8^ e-learning in the discipline of educational research,^9^ information systems and design fields^10^ and design of health informatics technologies.^11^ Some of these authors offer guidelines or frameworks for undertaking workshops as a research method in order to promote research rigour.^7 8 10^ Thoring *et al*^10^ actually offer guidelines for *evaluating* workshops as a research method, but in doing so identify issues relevant to undertaking workshops as a research method. Other authors highlight the variety of ways of conducting workshops and use this to offer guidance on organising workshops as a research activity,^11^ reflect on the value of workshops as a research method^9^ or present a case of a workshop used as a research method with methodological reflection.^8^ In health research, a recent article explores the epistemology of workshops as a research method alongside offering a practical guide to undertaking them.^4^ Trettin *et al*^4^ discuss workshops in the context of phenomenological-hermeneutic methodology which focuses on understanding lived experiences and sharing interpretations. The reflections below are based on this methodological literature. The interpretation of the literature has been shaped by the background and experience of the author of this paper who is a health services researcher who has written extensively about research methods—including developing complex interventions where workshops are common—and has led or participated in workshops undertaken both as a research method and stakeholder involvement in research.

The reflections below are offered as a complement to research approaches and fields that have used workshops extensively for decades, in particular, participatory research and design research. Indeed, the methodological literature drawn on acknowledges and references the literature specific to these approaches and fields while also identifying the gap relating to workshops as a research method in their own fields.^4 9 12^ The reflections are also a complement to specific approaches to workshops within research, such as the World Café method which aims to facilitate change within a community as well as explore a health topic.^13^ The reflections below are for health services researchers, clinical researchers and public health researchers who wish to use workshops without necessarily grounding them in participatory, design or community empowerment research.

## Reflections

### Definition of a workshop

Although Ørngreen and Levinsen^9^ found a lot written about workshops, they found much less written about workshops as a research method. They found that a workshop is weakly defined from an academic perspective.^9^ The definition of a workshop that they propose is, ‘an arranged event of limited duration, where participants from a similar domain meet, and is conducted by people with experience of the domain with an intention to promote genuine participation’.^9^

### Rationale for using workshops

Workshops target diverse stakeholders such as policy makers, service providers, clinicians, patients, carers, charities and members of the public so that interventions/products/strategies can be designed that meet all users’ needs.^7 11^ Some researchers propose that they complement other qualitative methods such as non-participant observation, focus groups and interviews because they enhance engagement,^7^ explore solutions to complex problems,^7^ generate ideas,^4^ encourage participants to learn from each other,^4^ offer deeper insights due to collaborative engagement^4^ and give voice to stakeholders as active participants rather than simply a source of information.^11^ Workshops complement qualitative interviews which can offer in-depth accounts of experiences and observation which can offer detailed descriptions of context.

### Key issues to consider when designing and reporting workshops

Workshops vary depending on their purpose and topic.^10^ Key aspects of workshops are explored in tables1^3^. The tables can be used to design a workshop or set of workshops. They can also be used to guide the reporting of a workshop in an academic publication to facilitate transparency and allow others to make judgements about the quality of the workshop for its intended aim. The tables are written for designing a workshop but a simple change of language makes them equally relevant to writing a peer-reviewed journal article that focuses only on workshops or includes workshops within a multiple or mixed methods publication.^7^ The information is displayed in three tables rather than one to aid readability.

**Table 1:** Key aspects of workshops as a research method (part 1)

Aspect	Insights from methodological literature
Planning and preparation	Plan a workshop, including whether some participants need preparation, training or materials before participating in the workshop.^7 8 11^ Researchers may explicitly ask participants to think about issues before attending.^8^
Aim or focus	Identify the focus of the workshop or the series of workshops (and the aim of each workshop within the series).^7 10 11^ In a series, each workshop can have a different aim. This aim is communicated to participants in invitations to participate in the workshop and at the beginning of a workshop.^4^
Methodological and conceptual frameworks	Reflect on whether methodological frameworks are relevant eg,participatory approaches,^8 11^ co-creation,^7^ design of technologies,^11^ co-design,^11^ user centred design,^11^ or participatory action research.^12^ Conceptual frameworks such as behaviour change theories or decision support frameworks^11^ may also be relevant.
Facilitator	Workshops benefit from a facilitator who is experienced in engaging and motivating a group to discuss their ideas.^7^ A facilitator requires advanced communication skills such as active listening and the ability to gain the trust of the group. A facilitator may be a researcher or an external expert facilitator.^7^ Be clear about the role of a facilitator.^4^ Consider the background of the facilitator and how this might affect the group dynamics.^11^
Process of selection of participants	Consider how participants will be selected,^7 11^ the roles of each stakeholder within the workshop^10^ and whether each workshop should include heterogeneous participants or only one type of participant (eg,patient).^11^ Workshop participants are usually heterogeneous.
Number of workshops	Consider how many workshops are needed. In some research, a single workshop may be required and in others multiple workshops. In a systematic review, 10 studies used a single workshop and 52 studies used multiple workshops.^11^ Hu^8^ describes a series of workshops in their case. In some studies, it may not be possible to identify the exact number of workshops needed if an intervention is being developed iteratively.^4^
Size of workshop	Consider how many participants need to attend each workshop. Size can vary, for example, workshops varied from 4 to 47 participants in a systematic review.^11^ A large workshop may also break participants up into smaller groups for activities, and the size of these smaller groups will also need consideration.^7^

**Table 2:** Key aspects of workshops as a research method (part 2)

Aspect	Insights from methodological literature
Duration	The length of each workshop can vary from 1 hour to over 1 day.^11^ Only 63% of 62 studies reported the duration of a workshop.^11^ The duration of each task or activity within a workshop also needs to be considered.^7^
Mode of delivery	Consider whether the workshop will take place online or in person, or a combination of the two over a series of workshops. Research had to move from in-person to online during the recent international COVID-19 pandemic, including workshops. Benson *et al*^12^ offer specific guidance on this context and Hu^8^ describes their experience of doing this. Online workshops may help or hinder inclusivity, for example, include people who do not want to travel or are unable to travel to a venue, or exclude people who are digitally challenged.
Location and space	Workshops can take place at a Research Institution, participants’ everyday context, or in a neutral setting.^7^ Enough space will be needed for breakout discussions. Consider how to arrange the space within the venue.^7^
Atmosphere	Think about creating a relaxed environment with coffee, snacks etc.^7 11^
Structure	Plan a structure for each workshop.^4^ There can be different phases of the workshop, for example, presentations by the research team or patients, whole group discussions, small group discussions and activities. Similarly, describe the structure of a set of workshops. Perhaps the first workshop focuses on exploration of the problem, the second on ideation of the intervention, the third on reflection of detailed components of the intervention prototype.^11^ Hu^8^ describes the structure of 8 workshops in their study.
Activities and their order	Consider the range of activities to be undertaken. For example, an ice-breaker to establish common ground between participants, creative techniques such as role play or scenarios, brainstorming using post-it notes or flipcharts, creating personas or vignettes, journey mapping of a patient through a service, storyboards of clinical processes,^9 11^ whole group and small group discussions,^11^ developing mock-up models or prototypes of an intervention for discussion,^11^ ranking exercises.^12^
Materials	Materials may need to be prepared or be available for use, for example, PowerPoint slides, post-it notes, vignettes on paper.

**Table 3:** Key aspects of workshops as a research method (part 3)

Power imbalances	Plan ways of addressing power imbalances between participants, and between participants and the researchers. This is essential to participatory research but important outside this context too. It can be challenging. Researchers can consider sharing planning tasks and decisions with participants,^8^ spending time on icebreakers and introductions,^4^ informing participants about boundaries and etiquette^9 12^ and having a good facilitator.^7^
Inclusion	Consider how inclusion will be addressed.^11^ How will diverse members of a community be recruited (eg, using community link workers?), will interpreters be needed? Will materials need to be translated or written in a way that is accessible to people with learning disabilities? Is the venue accessible? Does the food attend to cultural needs? Describe these issues in publications because they tend to be missing.^11^
Financial incentives or expenses	Consider whether some participants need payment for travel and for their time^12^ and the costs of the venue and refreshments.^4^
Data collection	Consider how to collect data.^4^ Examples include flipcharts, whiteboards or digital whiteboards in online workshops, interactive online resources within online workshops, post-its, audio-recording, video-recording, scribes who take notes and maps or diagrams produced by participants. The majority of workshops in 62 studies were audio or videotaped.^11^
Analysis	Recordings can be transcribed and analysed using a variety of approaches. These include thematic analysis or content analysis.^7 8 11 12^ Notes, post-it notes and flipcharts can be analysed using content analysis.^7 9^ Other approaches to analysis may be needed for visual materials produced by the participants.^8^
Publishing	It is usual to publish the results of a research method so publication would be expected. Workshops may be part of a multiple or mixed methods study,^9^,^11^ so reporting may occur alongside other methods.
Evaluation	Ozkaynak *et al*^11^ describe how only 7/62 workshop studies reported evaluation. Benson *et al*^12^ did formal evaluation of their workshop using an online survey. Thoring *et al*^10^ focus only on the evaluation of workshops and offer a framework for doing this. Researchers do not evaluate focus groups and interviews, so it is unusual to evaluate a research method. For workshops, evaluation may occur as a reflection on the quality of the research method for the intended purpose, for example, who attended, who was not in the room,^12^ how the mode of delivery affected findings, level of engagement. If undertaking a series of workshops, asking participants for reflection on early workshops can help to improve later ones.^7 8^ This way, participants can shape activities undertaken in later workshops.^8^ As a qualitative method, researchers can practise reflexivity during and after workshops.^4^

## Conclusion

Workshops as a research method aim to produce reliable and valid data about the issue being researched.^9^ Researchers need to decide if their planned workshop is a research method or stakeholder involvement in research. If it is a research method, then they should seek appropriate ethical review and report the key aspects described earlier to enhance reproducibility and allow others to assess the quality of the research.
