# Supplementary material for: Workshops as a qualitative research method in health research
Source: BMJ Open. 2025 Sep 26;15(9):e106459. doi: 10.1136/bmjopen-2025-106459 (PMC12481299; doi:10.1136/bmjopen-2025-106459)
Supplement: Supplementary file 1 [file bmjopen-2025-106459.reviewer_comments.pdf]

PEER REVIEW HISTORY

BMJ Open publishes all reviews undertaken for accepted manuscripts. Reviewers are asked to complete a checklist review form and are provided with free text boxes to elaborate on their assessment. These free text comments are reproduced below.

ARTICLE DETAILS

Title (Provisional)

Workshops as a qualitative research method in health research

Authors

O'Cathain, Alicia

VERSION 1 - REVIEW

**Reviewer**	**1**
**Name**	**Strange, Heather**
**Affiliation**	**Cardiff University, Centre for Trials Research**
**Date**	**26-Aug-2025**
**COI**	**None**

Thank you for inviting me to review this paper. It is clearly written, well-structured, and addresses an interesting, timely and important topic for health researchers to consider. It will be a useful resource, particularly for clinicians and/or researchers without substantial training in qualitative research methods. The table in particular is excellent, and has already helped me think upcoming workshop plans through more systematically – thank you! The current manuscript is very publishable but could benefit from a few very minor revisions.

The sentence on page 2, lines 9-13 which starts 'for example, researchers might use consensus methods' needs to be edited for clarity.

Page 2, lines 24-27: could include some information/description of how data may be collected digitally - e.g. via group chats, digital whiteboards etc. - and how this may offer increased opportunities for participant anonymity/inclusivity.

P3, line 48 – 'users who can be active participants' rather than simply 'active' sounds a little clearer

It could be worth considering whether other methods of analysis beyond thematic and content could be employed – would it be better to say such methods are most commonly, or typically used by researchers?

Within the conclusion, it would be helpful to briefly reflect on/clarify the limitations of workshops as a research method – what kind of research questions they are/are not appropriate for, what type of data they can/cannot produce (in-depth accounts of experience, detailed description of implementation contexts etc.)

**Reviewer**	**2**
**Name**	**Carrigan, Ann**
**Affiliation**	**The University of Sydney Faculty of Medicine and Health**
**Date**	**29-Aug-2025**
**COI**	**None**

Overall comments

The issues raised by the article are important to BMJ Open's broad and international readership that includes patients, researchers, policy makers, health professionals, and doctors of all disciplines.

The article is interesting and offers novel insights that have not been sufficiently considered in the existing published literature.

The article is well written and the content clearly presented. The message is clear but I have provided some recommendations below.

A revised version of article will help medical researchers, patients or related groups of readers to make better decisions.

This is a well written and important commentary that provides guidance for researcher to use workshops as a research method. I agree that more clarity is needed around the definition of a workshop and when to use them, from an academic perspective.

In the research community, I think there is confusion between what constitutes a focus group compared to a workshop, and sometimes these terms are used interchangeably. Consider differentiating these two methods early in the introduction. Although there is a section on focus groups on P2, the distinction is not clear to me. Ie; don't workshops also constrain who can attend, numbers, length of discussion?

Minor comments

P1 line 46: I disagree with the statement that "The term workshop is used outside the context of research…" as this is not my experience. There are many papers that cite workshops as part of their research methodologies. This needs to be toned down.

P2 line 12; this sentence does not make sense. Is there a word missing?

P2 line 15: "it is also the case that claiming them…" – are you referring to workshops or focus groups here? This needs clarification as the last sentence is long and confusing.

P2 line 25; data collection may also include flipcharts, whiteboards, interactive online resources.

P2 line 27; I am not sure I agree with the statement “a publication may mirror that of a qualitative study”. There are well-established papers that consider workshops as part of qual research. If you are saying that these are not qual because they have not been cited in “well-known research methods books”, this needs to be clarified as my understanding is that workshops are a qual data collection method, analysed formally, and mentioned in some resources. e.g. Hennink, M., Bailey, A., & Hutter, I. (2020). Qualitative Research Methods.

P3 Line 45: I would argue that workshops also “enhance engagement, explore solutions to complex problems and give voice to users.” I think this speaks again to the confusion about whether researchers are conducting workshops or focus groups. Ie they may call them a workshop when they are indeed focus groups! (or vice versa).

P3 Line 52- this section and subsequent table are important and would be very helpful for the research community.

VERSION 1 - AUTHOR RESPONSE

**Reviewer: 1**<br>Thank you for inviting me to review this paper. It is clearly written, well-structured, and addresses an interesting, timely and important topic for health researchers to consider. It will be a useful resource, particularly for clinicians and/or researchers without substantial training in qualitative research methods. The table in particular is excellent, and has already helped me think upcoming workshop plans through more systematically – thank you! The current manuscript is very publishable but could benefit from a few very minor revisions.	Many thanks for this positive feedback and the recommendations for improving the paper.
The sentence on page 2, lines 9-13 which starts ‘for example, researchers might use consensus methods’ needs to be edited for clarity.	I have edited this sentence
Page 2, lines 24-27: could include some information/description of how data may be collected digitally - e.g. via group chats, digital whiteboards etc. - and how this may offer increased opportunities for participant anonymity/inclusivity.	I have now included this in the table under ‘mode of delivery’ and ‘data collection’

P3, line 48 – 'users who can be active participants' rather than simply 'active' sounds a little clearer	I have edited this as recommended
It could be worth considering whether other methods of analysis beyond thematic and content could be employed – would it be better to say such methods are most commonly, or typically used by researchers?	I have edited the text to indicate that analysis approaches can include these two approaches rather than imply that these are the only options available. I have also extended the 'analysis' section of the table to include other approaches
Within the conclusion, it would be helpful to briefly reflect on/clarify the limitations of workshops as a research method – what kind of research questions they are/are not appropriate for, what type of data they can/cannot produce (in-depth accounts of experience, detailed description of implementation contexts etc.)	This is a great idea. I thought this would fit better in the 'rationale for using workshops' section so have expanded this section so the strengths and limitations of workshops are clear.
**Reviewer: 2 Overall comments**<br><br>The issues raised by the article are important to BMJ Open's broad and international readership that includes patients, researchers, policy makers, health professionals, and doctors of all disciplines. The article is interesting and offers novel insights that have not been sufficiently considered in the existing published literature.<br><br>The article is well written and the content clearly presented. The message is clear but I have provided some recommendations below.<br><br>A revised version of article will help medical researchers, patients or related groups of readers to make better decisions.<br><br>This is a well written and important commentary that provides guidance for researcher to use workshops as a research method. I agree that more clarity is needed around the definition of a workshop and when to use them, from an academic perspective.	 <br>Many thanks for this positive feedback and the recommendations for improving the paper.

In the research community, I think there is confusion between what constitutes a focus group compared to a workshop, and sometimes these terms are used interchangeably. Consider differentiating these two methods early in the introduction. Although there is a section on focus groups on P2, the distinction is not clear to me. don't workshops also constrain who can attend, numbers, length of discussion?	I agree and have extended the section on focus groups in the introduction. Trettin and colleagues (2025) have published on workshops in the health sciences recently and describe the differences between workshops and focus groups really well. So I have referenced their work.
**Minor comments**<br>P1 line 46: I disagree with the statement that "The term workshop is used outside the context of research…" as this is not my experience. There are many papers that cite workshops as part of their research methodologies. This needs to be toned down.	I have deleted this sentence because it does not communicate clearly the point I was trying to make. The point I was trying to make was that it is a term used in common parlance in the context of training e.g. I have been invited to a workshop on performance managing staff to enhance my skills as a manager.<br><br>I agree with the point you make about toning down my language and have made edits to address this (see later comment)
P2 line 12; this sentence does not make sense. Is there a word missing?	There were a couple of random words in this sentence. I have corrected this now.
P2 line 15: "it is also the case that claiming them…" – are you referring to workshops or focus groups here? This needs clarification as the last sentence is long and confusing.	I meant workshops so have corrected this now.
P2 line 25; data collection may also include flipcharts, whiteboards, interactive online resources.	Absolutely. I have added a section to the Table addressing data collection
P2 line 27; I am not sure I agree with the statement "a publication may mirror that of a qualitative study". There are well-established papers that consider workshops as part of qual research. If you	I agree and have made edits throughout to remove this confusion e.g. stating now that researchers would write a

are saying that these are not qual because they have not been cited in “well-known research methods books”, this needs to be clarified as my understanding is that workshops are a qual data collection method, analysed formally, and mentioned in some resources. e.g. Hennink, M., Bailey, A., & Hutter, I. (2020). Qualitative Research Methods.	qualitative research article rather than ‘mirror one’, saying that well known research methods books do not *always* include workshops as a research method.
P3 Line 45: I would argue that workshops also “enhance engagement, explore solutions to complex problems and give voice to users.” I think this speaks again to the confusion about whether researchers are conducting workshops or focus groups. Ie they may call them a workshop when they are indeed focus groups! (or vice versa).	I think the additional text about differentiating focus groups from workshops will help to reduce confusion
P3 Line 52- this section and subsequent table are important and would be very helpful for the research community.	Thank you

VERSION 2 - REVIEW

Reviewer 2

Name Carrigan, Ann

Affiliation The University of Sydney Faculty of Medicine and Health

Date 11-Sep-2025

COI

The author has done a great job in addressing my comments and suggestions. No further comments from me.
